# Understanding whose births get registered: a cross sectional study in Bauchi and Cross River states, Nigeria

**DOI:** 10.1186/s13104-015-1026-y

**Published:** 2015-03-13

**Authors:** Atam E Adi, Tukur Abdu, Amir Khan, Musa Haruna Rashid, Ubi E Ebri, Anne Cockcroft, Neil Andersson

**Affiliations:** CIET Trust, 71 Oxford Road, 2196 Johannesburg, Saxonwold South Africa; Institute of Geography and urban regional planning, University of Peshawar, Peshawar, Pakistan; Director of Vital Statistics, National Population Commission, Bauchi, Bauchi State Nigeria; Head of Department Vital Statistics, National population Commission, Calabar, Cross River State Nigeria; CIET Trust Botswana, PO Box 1240, Gaborone, Botswana; CIET-PRAM, Department of Family Medicine, McGill University, Montreal, Canada

**Keywords:** Birth registration, Antenatal care, Socio-economic status, Nigeria

## Abstract

**Background:**

It is a recognized child right to acquire a name and a nationality, and birth registration may be necessary to allow access to services, but the level of birth registration is low in Nigeria. A household survey about management of childhood illnesses provided an opportunity to examine actionable determinants of birth registration of children in Bauchi and Cross River states of Nigeria.

**Methods:**

Trained field teams visited households in a stratified random cluster sample of 90 enumeration areas in each state. They administered a questionnaire to women 14–49 years old which included questions about birth registration of their children 0–47 months old and about socio-economic and other factors potentially related to birth registration, including education of the parents, poverty (food sufficiency), marital status of the mother, maternal antenatal care and place of delivery of the last pregnancy. Bivariate then multivariate analysis examined associations with birth registration. Facilitators later conducted separate male and female focus group discussions in the same 90 communities in each state, discussing the reasons for the findings about levels of birth registration.

**Results:**

Nearly half (45%) of 8602 children in Cross River State and only a fifth (19%) of 9837 in Bauchi State had birth certificates (seen or unseen). In both states, children whose mothers attended antenatal care and who delivered in a government health facility in their last pregnancy were more likely to have a birth certificate, as were children of more educated parents, from less poor households, and from urban communities. Focus group discussions revealed that many people did not know about birth certificates or where to get them, and parents were discouraged from getting birth certificates because of the unofficial payments involved.

**Conclusion:**

There are low levels of birth registration in Bauchi and Cross River states, particularly among disadvantaged households. As a result of this study, both states have planned interventions to increase birth registration, including closer collaboration between the National Population Commissions and state health services.

## Background

Birth registration of children may be required for access to health, education and other services in some places; it allows accurate estimation of age (for example in relation to military service or marriage); it helps in reuniting separated children and parents; and it can provide important population information for planning of services [1]. Birth registration is recognized as a right of all children [2]. In 2013, based on figures up to 2011, UNICEF estimated that only 65% of the world’s children are registered: 38% are registered in the least developed countries, and 44% are registered in countries of sub-Saharan Africa [3].

Birth registration levels in developing countries have been reported to be related to demand factors such as socio-economic status, parental education, religion, ethnicity, maternal age and marital status [1,4-7], as well as supply factors such as geographic distance to registration facilities [8], and mixed factors such as urban/rural location, attendance for antenatal care, place of birth, and skilled birth attendance [1,4,5].

In Nigeria, the National Population Commission (NPopC) is the body responsible for the national vital registration system, including birth registration [9]. Births can be registered at NPopC centres, located in government health facilities and in Local Government Authority (LGA) centres. To register a birth not occurring in a government facility, an affidavit about place of birth is required from the LGA. Information recorded on the certificate includes the child’s sex, name, date and place of birth, and the names of the mother and father. Birth registration is compulsory in Nigeria, under the Births, Deaths etc. (compulsory registration) Act number 69 of 1992 [10]. In at least some places, a birth certificate may be a requirement for school enrollment in Nigeria [11] and it is said to be required for obtaining a job in the public sector [10].

Despite birth registration being compulsory, levels in Nigeria remain low. The 2013 Demographic and Health Survey (DHS) reported that 30% of children less than five years old had their births registered [12]. Multiple Indicator Cluster Surveys (MICS) have reported birth registration rates among Nigerian children less than five years old of 23% in 2007 [13] and 42% in 2011 [14]. In 2013, 57% of birth registrations took place at a NPopC centres, 13% at LGA offices, and 22% at private clinics or hospitals [12].

As part of a household survey on childhood illnesses and their management, we examined the level of birth registration and the socio-economic and other factors potentially related to birth registration among children aged 0–47 months old in Cross River and Bauchi states of Nigeria. Bauchi State in the North East region is predominantly Muslim with *Hausa* and *Fulani* cultures, including polygamy and extended family households, while Cross River State in the South is predominantly Christian with a majority of *Efik* people and more typically nuclear families.

## Methods

In 2011, a household survey on childhood illnesses and their management was part of a programme to support evidence-based planning of primary health services in Bauchi and Cross River states of Nigeria [15,16]. The random cluster sample of enumeration areas, stratified by urban/rural location, from the 2006 census comprised 90 clusters in each state. In each cluster, interviewers visited contiguous households radiating from a random starting point, covering all children in each household under four years old, until they had collected data on approximately 100 children under four years old.

Trained female interviewers administered a questionnaire to mothers aged 14–49 years of children aged less than four years. In Bauchi it is not possible for males to interview women and in Cross River female interviewers are preferred. The age range of birth to four years was chosen because an aim of the overall work was to examine management of childhood illnesses among children up to three years old. The field teams also collected information from each household about demographics and socio-economic status, and recorded information from key informants in each community about access to services.

Three months after the household survey, after initial analysis of the findings from this survey, trained teams returned to the same communities to conduct separate male and female focus group discussions. In each group of 8–10 parents from the community, a facilitator used a guide to share information from the household survey and to encourage discussion around the findings, while a reporter took notes. The discussions took place in the local language but the final reports from the discussions, agreed between the facilitator and the reporter, were in English.

### Analysis

Different operators entered the data twice with validation to minimize keystroke errors using EpiInfo [17]. Analysis relied on CIETmap [18], an open source software with a windows-like interface with the popular statistical programming language R. Analysis weighted all estimates proportional to the population in each state, allowing for differences between the urban/rural balance in the sample and in the census population.

Bivariate analysis examined potential associations with birth registration. Subsequent multivariate analysis used the Mantel Haenszel procedure [19], adjusted for clustering [20]. Saturated multivariate models included variables significant in the bivariate analysis. We used backward deletion to arrive at the final multivariate models in which all variables were significantly associated with the outcome. We present associations using adjusted Odds Ratios (aOR) with the cluster adjusted 95% confidence interval (CIca). In the event of interaction in multivariate models, we undertook separate modeling for the two levels of the interacting variable; for this reason, in Bauchi state we prepared separate models for rural and urban residence.

We conducted separate analyses for the two states. There is no intention for the two states together to be representative of the situation across Nigeria, and the data collection and analysis is part of a programme to support evidence-based planning of health services at state level [15,16].

The analysis included children aged 0–47 months whose mothers had been pregnant within the last two years. We categorized children as having a birth certificate if the mother reported their having one, whether or not she could show it. Potential determinants of birth registration included: sex of the child; education of the mother and father of the child (any formal education in Bauchi, junior secondary or above in Cross River); whether the mother had any antenatal care visits, and whether she delivered in a government health facility in her last pregnancy; whether the mother had an income of her own and was involved in the decision on how to spend it; and how many children the mother had (two or less v more than two). At household level, potential determinants included whether the household had enough food in the last one week (as an indicator of absolute poverty) and sex of the household head. At community level, potential determinants included urban or rural location and whether there was an active village health committee.

Two coauthors, AA and AC, conducted a thematic analysis of the focus group reports to identify common themes of why parents do not register the birth of children and what could help to increase birth registration.

### Ethical approval

The Ministry of Health in each state gave ethical approval for the study. In addition, field team leaders sought consent for the survey from leaders in each community, and interviewers sought verbal consent from the head of each household, as well as from each individual respondent. Interviewers did not record any names or identifying information and were trained not to proceed with any interview unless they could do so without being overheard.

## Results

Interviewers collected information about 18,439 children aged 0–47 months old: 8,602 from Cross River and 9,837 from Bauchi.

Less than one half of the children in Cross River (45%) and about one fifth in Bauchi (19%) had a birth certificate (Table 1) as reported by their mothers. Table 1 shows weighted frequencies for characteristics potentially related to birth registration. Few children in either state were from households without enough food in the last week (an indicator of serious poverty). Most mothers had attended for antenatal care (ANC) in a government health facility during the last pregnancy, but few mothers delivered their last child in a government health facility. The great majority of mothers in Cross River were married or cohabiting and almost all in Bauchi were married.

**Table 1:** **Characteristics of children aged 0**–**47 months with mothers 14**–**49 years who were pregnant in the last two years**

**Factors**	**Cross River state**	**Bauchi state**
	**Weighted percent** **(fraction)**	**Weighted percent** **(fraction)**
With birth certificate	44.9(3973/8425)	18.9(1790/9670)
Male	49.4(4214/8600)	50.7(4958/9836)
Mother married or co-habiting	89.2(7646/8562)	99.2(9731/9817)
Mother attended had ANC in a government health facility in her last pregnancy	73.8(6451/8533)	69.8(6767/9826)
Mother delivered in a government health facility in her last pregnancy	32.5(2770/8464)	16.9(1581/9707)
Mothers has < 2 children	46.1(3913/8598)	35.3(3453/9823)
Mother has an income and decides on how to spend it	46.7(3990/8590)	51.4(5128/9816)
Mother has some formal education	94.5(8030/8544)	19.1(1960/9830)
Mother has junior or higher education	62.4(5327/8544)	8.7(830/9830)
Father has some formal education	95.6(7841/8244)	34.2(3407/9732)
Father has junior or higher education	72.1(5900/8244)	25.0(2468/9732)
From female headed households	14.3(1232/8576)	0.4(40/9789)
From households with enough food in the last week	80.8(6860/8544)	89.8(8859/9758)
From urban area	32.2(2802/8602)	18.9(1600/9837)
From community with a government health facility	74.7(6439/8405)	58.3(6018/9789)
From community with active village health development committee	55.6(4384/8122)	16.8(2223/9753)

**Figure 1:**
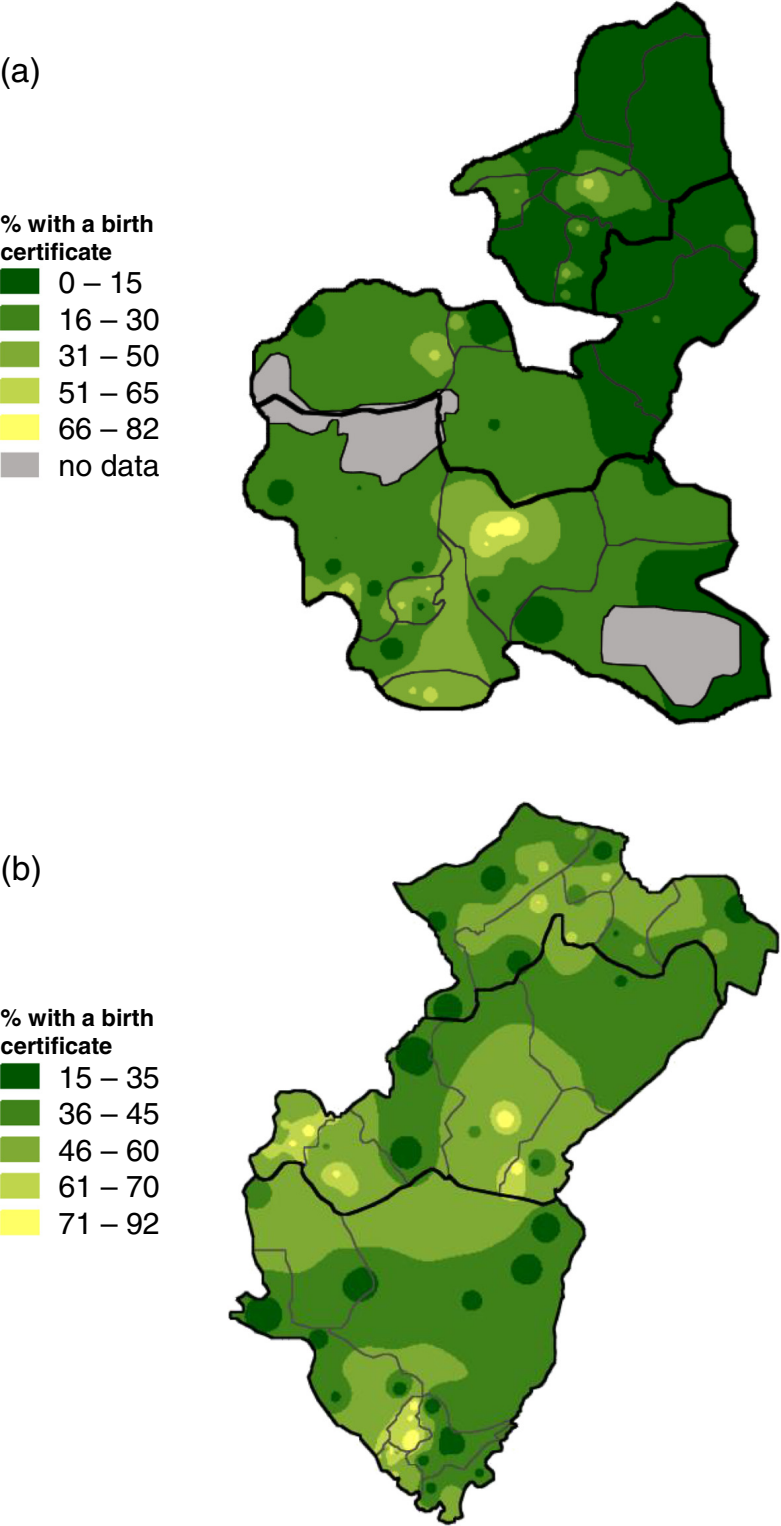
**Birth registration variation across Bauchi and Cross River states.** Detailed legend: The population-weighted raster maps show the proportion of children aged 0–47 months old with a birth certificate across **(a)** Bauchi and **(b)** Cross River states.

Figure 1 shows the geographic variation in birth registration. In Bauchi state, birth certification was more common in the southern part of the state. There was no clear geographic variation in Cross River state.

Table 2 shows bivariate associations with birth registration in each state. The factors significantly associated with birth registration in this bivariate analysis were very much the same in the two states. In both states birth registration was more likely in urban areas and among children whose mothers attended ANC and delivered in a health facility for their last pregnancy, as well as among children with more educated parents and from less poor households. In Cross River, children of married or cohabiting mothers were slightly more likely to have had their births registered, but the association was not significant at the 5% level. In Bauchi, children of married mothers (99%) were less likely to have had their births registered than the 1% whose mothers were not married or cohabiting.

**Table 2:** **Bivariate associations between birth registration and potential determinants among children aged 0**–**47 months** (**with mothers aged 14**–**49 years pregnant in the last 2 years**

**Factors**	**Cross River state**	**Bauchi state**
	**OR** **(95%** **CIca)**	**OR** **(95%** **CIca)**
From urban area	**1.79 ** **(1.33 - ** **2.42)**	**4.80** **(2.82** - **8.16)**
Male	1.03 (0.95 - 1.11)	1.02 (0.91 - 1.13)
Mother married or co-habiting	1.11 (0.92-1.33)	**0.54 ** **(0.32-** **0.92)**
Mother attended antenatal care in a government health facility in her last pregnancy	**2.53** **(2.18 - ** **2.93)**	**4.26 ** **(3.00 - ** **6.04)**
Mother delivered in a government health facility in her last pregnancy	**2.31** **(1.95 - ** **2.74)**	**4.41** **(3.31 - ** **5.87)**
From household with enough food in the last week	**1.39** **(1.20 - ** **1.60)**	1.29 (0.96 - 1.72)
Mother has less than two children	1.08 (0.96 - 1.20)	0.99 (0.85 - 1.16)
Mother has an income and is involved in decision on how to spend it	1.09 (0.96 - 1.23)	1.04 (0.86 - 1.25)
Mother has some formal education	**1.88** **(1.40 - ** **2.53)**	**4.77** **(3.73 - ** **6.11)**
Mother has junior secondary or higher education	**2.14** **(1.84 - ** **2.49)**	**6.69 ** **(4.79 - ** **9.36)**
Father has some formal education	**2.04 ** **(1.60 - ** **2.60)**	**4.32 ** **(3.30 - ** **5.66)**
Father has junior secondary or higher education	**1.76 ** **(1.54 - ** **2.01)**	**4.72 ** **(3.63 - ** **6.13)**
From a female headed household	0.89 (0.76 - 1.04)	1.17 (0.58 - 2.37)
From community with an active village health development committee	0.89 (0.67 - 1.20)	1.32 (0.76 - 2.29)
From community with a government health facility within the community	1.32 (0.90-1.94)	**1.92 ** **(1.15-** **3.21)**

**Bold** font indicates associations significant at the 5% level.

Table 3 shows the final multivariate models. We included variables significant in bivariate analysis in the initial multivariate models. Due to an interaction with the variable for urban/rural location, we developed separate models for urban and rural households in Bauchi state. The results of all three final models show that the average child was more likely to have a birth certificate if the mother delivered her last pregnancy in a government health facility and if the mother had more education. Father’s education and mother’s antenatal care were significant factors in Cross River and in rural areas of Bauchi. In addition, in Cross River children from urban areas and from better off households were more likely to have a birth certificate.

**Table 3:** **Final multivariate models of factors associated with birth registration in Cross River and Bauchi states**

**Factors**	**Crude OR**	**Wt OR**	**Cl adj 95% ** **CI**
**Cross River state** (n = 7808)			
Mother had government antenatal care	2.47	1.75	1.51-2.03
Mother delivered last pregnancy in a government health facility	2.30	1.73	1.47-2.03
Mother with junior secondary or higher education	2.10	1.59	1.38-1.83
Father with junior secondary or higher education	1.78	1.16	1.03-1.32
Household with enough food in the last week	1.38	1.17	1.03-1.34
Urban household	1.80	1.55	1.17-2.04
**Bauchi state**			
*Urban (n = 1532)*			
Mother delivered last pregnancy in a government health facility	3.24	2.78	1.87-4.12
Mother with some formal education	2.35	1.81	1.33-2.46
*Rural (n = 7915)*			
Mother had government antenatal care	3.39	1.93	1.42-2.64
Mother delivered last pregnancy in a government health facility	3.04	1.98	1.40-2.80
Mother with some formal education	4.57	2.31	1.61-3.31
Father with some formal education	3.99	2.25	1.68-3.02

### Views from focus groups

Many groups said that people did not know about birth certificates or where to get them.

*“We are in the village and do not know what all these things are.”* (female group, Bauchi).

Others did not see the point in getting a birth certificate.

*“Since we know the child’s birth date, why should we get a birth certificate?”* (Female group, Cross River).

Participants agreed that women not delivering in government health facilities were unlikely to get a birth certificate for the child.

*“Some women deliver in the church and it is only bibles and hymn books that are there, so they cannot have birth certificates.”* (Female group, Cross River).

A common complaint was that people have to pay to get birth certificates.

*“I took my child to collect a certificate and I was asked to pay Naira 1500 (US $9), I pleaded to pay Naira 1000 (US $6) but they refused.”* (Male group, Bauchi).

*“I paid Naira 300 (US $2) to get a certificate for my child. Is it fair?”* (Female group, Cross River).

Some said women were loath to request a birth certificate if they did not know the father’s name.

*“Some women do not know the names of their husbands” *(Female group, Cross River).

Focus group participants agreed people need to be made more aware about the importance of birth certificates. They suggested an important role for community leaders in raising awareness.

*“Community leaders should tell the men to encourage their wives to go to the health center and deliver so that birth certificates can be obtained easily.”* (Female group, Bauchi).

*“The community chief should impose a law.”* (Female group, Cross River).

## Discussion

In this study, the level of birth registration in 2011 among children less than four years old was low in Cross River at 45% and very low in Bauchi at 19%. Despite the higher overall level of birth registration in Cross River, the factors associated with birth registration were similar in the two states. Children were more likely to have their birth registered if they came from urban households, their parents were more educated, and if their mothers had attended for antenatal care and delivered in a government health facility. In Cross River, less-poor households were more likely to register births.

A strength of this study is that it is based on quite large representative samples in each of the two states. Also, it includes qualitative data from focus groups in the same sites as the household survey, allowing exploration of the reasons behind the quantitative findings. A weakness of the study is that it did not collect data about some potential determinants of birth registration, in particular about supply factors such as distance to and features of the nearest facility where births could be registered. As it is a cross-sectional study, we can only describe associations rather than draw conclusions about causality.

The levels of birth registration in this study can be compared with other surveys providing state-level estimates in Nigeria. The 2011 MICS reported birth registration levels among children less than five years old as 38% in Cross River and 6% in Bauchi [14]. The MICS 2011 figure for Bauchi is notably lower than that in our study and indeed lower than the figure for Bauchi in the 2007 MICS, which reported birth registration of 27% in Cross River and 23% in Bauchi [13]. The 2013 DHS reported birth registration levels among children less than five years old as 22% in Cross River and 14% in Bauchi [12]. Our estimate of birth registration for Bauchi is similar to that of the 2013 DHS. It is not clear why the DHS estimate for Cross River is lower than ours. The DHS sample included only 536 children in Cross River.

The demand factors we found associated with birth registration are similar to those reported previously. A UNICEF report, based on data from DHS and MICS from a number of countries, concluded that poverty and low levels of maternal education and knowledge were associated with failure to register births [1]. Reports from Latin America, based on analysis of DHS data, found that under-registration of births was associated with poverty, young maternal age, poor maternal education and indigenous origin [4,5]. Small studies in areas of Kenya and Nigeria reported associations between low household income and low maternal education and under-registration of births [6,7]. Our finding that children from rural areas were less likely to have their births registered also agrees with findings from elsewhere [1,4,5] and probably reflects less access to registration facilities in rural areas. Access is clearly a factor; a study in three countries in Latin America measured distance to a birth registration facility and noted that registration was less likely when the facility was further away, taking other factors into account [8]. In our study, in Bauchi birth registration was more likely if there was a government health facility within the community (Table 2), but this association did not remain significant when other factors were taken into account, including whether the mother attended for government antenatal care and delivered in a government health facility. The association of birth registration with attendance for antenatal care and institutional delivery or skilled birth attendance has been noted consistently [1,4,5]. Since government health facilities are one of the places where birth registration takes place in Nigeria, it is not surprising that mothers with more contact with these facilities, particularly for delivery, were more likely to register their children’s births.

The focus group discussions in this study provided some insights to supplement the quantitative survey findings. Participants in the groups agreed that parents often do not register a birth if it does not take place in a government health facility. They do not see enough reason to make the effort required to go and register the birth. Quite a common complaint in the focus groups was about having to pay for birth certificates, which are theoretically provided free of charge. Demands for such payments may well deter birth registration. Previously, a “fine” was payable for birth registration more than 60 days after birth, although this fine was not charged in practice. Birth registration is now free of charge, by law, up to the age of 17 years. The household survey did not ask about payment for birth certificates, so we do not know how frequently people paid, or how much they paid. Focus group participants mentioned amounts equivalent to several US dollars. This is substantial in a country where over half the population live on less than US $1.25 per day.

Birth registration is a function of local government and Nigerian authors have noted important constraints of local government, including lack of financial and human capacities, lack of input into resource allocation decisions, lack of adequately qualified personnel, and widespread corruption [21].

UNICEF has described strategic approaches for strengthening birth registration in Africa [22]. A recommended approach is to integrate birth registration more closely with health services [23]. An increase in birth registration in Ghana between 2003/4 and 2008 has been attributed to a package of interventions, including incorporating birth registration into child health weeks, training community health workers to register births, and instructing health workers to register children during child health campaigns [24]. Based on the findings from the study reported here, the NPopC branches in Cross River and Bauchi states have taken and are taking action to improve birth registration, in collaboration with the state Ministries of Health. In late 2011, the government in Cross River began training health workers in government facilities to act as registrars and issue permanent birth certificates. The NPopC in Cross River also intends to train teachers in primary schools as birth registrars, so that any child enrolling without a birth certificate can be registered at the school, and to set up programmes of advocacy for birth registration with local government staff, targeting LGAs with low birth registration during health weeks, and providing extra staff during these weeks. In Bauchi, the NPopC plans to hold LGA workshops to improve birth registration coverage, and to partner with the state ministry of health to train health workers to register children in government health facilities. Further measurement of birth registration levels in the two states will be needed to indicate whether there has been improvement following the interventions by the state governments.

## Conclusion

This study found that low levels of birth registration were related to lack of contact with government health services during pregnancy and childbirth in both Cross River and Bauchi states and that more disadvantaged children (from rural areas, from the poorest households, and with less educated parents) were less likely to have their births registered. Both states have used the findings to put in place measures to increase birth registration, including increased collaboration between the NPopC and health services.

## Abbreviations

NPopC: National Population Commission
LGA: Local Government Authority
ANC: Antenatal Care
DHS: Demographic and Health Survey
MICS: Multiple Indicator Cluster Survey
